# Molecularly Adapted Antitumor Therapy for Newly Diagnosed Diffuse Large B-Cell Lymphoma: Two-Year Follow-Up Results

**DOI:** 10.3390/jcm15082983

**Published:** 2026-04-14

**Authors:** Marat Mingalimov, Elena Baryakh, Andrey Misyurin, Laura Kesaeva, Hasmik Mkrtchyan, Elena Misyurina, Mariia Orlova, Tatiana Tolstykh, Ekaterina Zotina, Liliia Shimanovskaia, Tatiana Chudnova, Diana Ivanova, Olga Kochneva, Kseniya Tsurkina, Dmitry Lebedev, Georgii Tyshkevich, Natalia Bekreneva, Viktoriia Basova, Mikhail Donskoy, Sergej Rodnikov, Ivan Abramov, Natalia Bodunova, Saida Gadzhieva, Tatiana Semina, Sergey Andreev, Inna Samsonova, Mariana Lysenko

**Affiliations:** 1 Moscow Clinical Science and Research Center 52, 123182 Moscow, Russia; baryakh_e_a@staff.sechenov.ru (E.B.); misyurina_e_n@staff.sechenov.ru (E.M.); tolstykh_t_n@staff.sechenov.ru (T.T.); zotina_e_n@staff.sechenov.ru (E.Z.); vagizova2016@list.ru (L.S.); chudnova_t_s@staff.sechenov.ru (T.C.); doc.ivanovadd@gmail.com (D.I.); kochneva_o_l@staff.sechenov.ru (O.K.); tsurkina_k@zdrav.mos.ru (K.T.); korsar666666@gmail.com (D.L.); natali-bek@yandex.ru (N.B.); s.e.r.g-666@inbox.ru (S.R.); andreevss@mos.ru (S.A.); samsonovaiv@mos.ru (I.S.); lysenkoma@mos.ru (M.L.); 2 Department of Hematology, I.M. Sechenov First Moscow State Medical University—Ministry of Health of Russia (Sechenov University), 119991 Moscow, Russia; orlova_m_s@student.sechenov.ru; 3 FSBEI FPE «Russian Medical Academy of Continuous Professional Education» of the Ministry of Healthcare of the Russian Federation, 125993 Moscow, Russia; 4 N.I. Vavilov Institute of General Genetics, 119991 Moscow, Russia; and@genetechnology.ru (A.M.); vvvbasovavvv@gmail.com (V.B.); 5 LLC «Genetechnology», 117437 Moscow, Russia; genlab@bk.ru (L.K.); hasm.mkrtchyan@gmail.com (H.M.); 6 FSBI FPE «Central State Medical Academy» of the Administrative Department of the President of the Russian Federation, 121359 Moscow, Russia; info@cgma.su; 7 JSC «European Medical Center», 129110 Moscow, Russia; donskoyma@yandex.ru; 8 SBIH Moscow Clinical Scientific and Practical Center named after A.S. Loginov of DHM Moscow, 111123 Moscow, Russia; abriv@bk.ru (I.A.); bodunovana@zdrav.mos.ru (N.B.); 9 Moscow Healthcare Department, 43 Oruzheynyy Pereulok, 127006 Moscow, Russia; gadzhievasm@mos.ru (S.G.); seminata@mos.ru (T.S.)

**Keywords:** diffuse large B-cell lymphoma, molecularly adapted therapy, R-CHOP-X, LymphGen, targeted sequencing, personalized medicine, progression-free survival, overall survival

## Abstract

**Background/Objectives:** Diffuse large B-cell lymphoma (DLBCL) is molecularly heterogeneous, and approximately 30-50% of patients fail to achieve cure with standard R-CHOP. Genotype-directed first-line therapy may improve outcomes by targeting subtype-specific oncogenic pathways. This study evaluated the feasibility, efficacy, and safety of a molecularly adapted R-CHOP-X strategy with two-year follow-up. **Methods:** In this single-center, prospective, non-randomized study conducted between September 2023 and the data cut-off (16 September 2025), 43 adults with newly diagnosed DLBCL (excluding high-grade B-cell lymphoma, primary immune-privileged, and primary mediastinal large B-cell lymphomas) underwent tumor genotyping using the LymphGen classification after targeted sequencing: a 19-gene Sanger panel (Cohort 1, n = 35) or an expanded 60-gene panel (Cohort 2, n = 8; proof-of-concept). All patients received one initial cycle of R-CHOP as bridge therapy pending molecular profiling results, followed by five cycles of R-CHOP-X, with the additional agent (vorinostat, acalabrutinib, decitabine, or lenalidomide) selected according to molecular subtype. Response was assessed by PET/CT per Lugano criteria; adverse events were graded per NCI CTCAE v5.0. **Results**: The overall study population was predominantly high-risk: 72% had an IPI of 3–5, 58% had stage III–IV disease, and 67% exhibited a non-GCB immunophenotype. Expansion from the 19-gene to the 60-gene panel reduced unclassifiable (NOS) cases from 34% to 12%. The overall response rate was 100% (43/43); complete response among patients completing therapy was 100% (35/35). At two years, overall survival was 92% (95% CI 83–100%) and progression-free survival was 94% (95% CI 86–100%). Two early relapses occurred (NOS and N1 subtypes), both resulting in death. Grade 3–4 neutropenia, thrombocytopenia, and anemia occurred in 26%, 12%, and 7% of patients, respectively; no dose reductions or treatment discontinuations were recorded. **Conclusions:** Molecularly adapted R-CHOP-X is feasible and associated with high response rates and favorable two-year survival in newly diagnosed DLBCL, comparing favorably with historical R-CHOP outcomes in high-risk populations. Expanded genomic panels substantially improve molecular classifiability. These findings warrant validation in larger, multicenter, randomized clinical trials.

## 1. Introduction

It is now well established that diffuse large B-cell lymphoma (DLBCL) comprises a biologically and clinically heterogeneous group of aggressive B-cell neoplasms. DLBCL is characterized by distinct molecular mechanisms of lymphomagenesis, variable prognosis, differential therapeutic sensitivity, and divergent clinical outcomes [1,2]. This heterogeneity is reflected not only in recent revisions of hematolymphoid tumor classifications—the 5th edition of the WHO classification [3] and the International Consensus Classification (ICC, 2022) [4]—but also in contemporary molecular stratification frameworks for DLBCL. Systems such as HMRN [5], LymphGen [6], and DLBclass [7] provide detailed genetic segregation of patients and carry direct prognostic and therapeutic implications for understanding lymphomagenesis, risk assessment, prediction of treatment response, and clinical outcome. It is important to emphasize that DLBCL is distinguished not only by molecular heterogeneity but also by its potentially curable nature. Accordingly, the primary objective of antitumor therapy remains the achievement of a definitive cure for patients with this aggressive B-cell malignancy [8].

Despite progress in diagnostics and therapy, DLBCL remains a major unresolved challenge in oncohematology, both scientifically and clinically. This is largely attributable to the high incidence of early relapse, the frequency of primary refractory disease, and substantial disease-related mortality.

Therapeutic management of newly diagnosed DLBCL remains a subject of debate. Currently, several conceptually distinct treatment strategies coexist: a risk-adapted approach, which selects the optimal immunochemotherapy based on progression risk strata; an response-adapted approach, which optimizes treatment according to dynamic assessments of therapeutic response; a molecularly agnostic approach, which applies uniform therapy irrespective of tumor molecular characteristics; and a molecularly oriented (targeted) approach, which aims to interrupt key aberrant signaling cascades of lymphomagenesis based on the tumor’s biological profile [9,10].

Notwithstanding this heterogeneity, the leading treatment paradigm for newly diagnosed DLBCL remains molecularly agnostic therapy, principally the programmed immunochemotherapy (ICT) regimen R-CHOP, which is endorsed by national and international clinical guidelines [11,12,13]. Nonetheless, most experts consider the outcomes achieved with this regimen to be suboptimal, motivating the search for innovative strategies to improve first-line treatment efficacy in de novo DLBCL. This is supported by data from systematic reviews indicating that approximately 30–50% of patients fail to be cured with R-CHOP, depending on disease stage and the constellation of prognostic factors. Importantly, the pattern of treatment failure is heterogeneous, with approximately 20% of cases representing primary refractory disease and about 30% relapsing after an initial complete remission [14]. Importantly, the International Prognostic Index and its modified versions lack sufficient predictive accuracy to reliably identify primary refractory disease at the time of diagnosis. Moreover, even the implementation of contemporary molecular classifiers has not eliminated the existing “blind spot” with regard to primary resistance to therapy. It has been shown that primary refractory disease is significantly enriched for mutations in genes such as *TP53* (34% vs. 15%) and *ARID1A* (21% vs. 7%); however, the authors emphasize that current molecular classification systems do not allow for reliable identification of such cases at treatment initiation [15]. In this context, it is essential to emphasize that R-CHOP failures are largely biologically driven. A considerable proportion of refractory disease is linked to high-risk molecular subgroups that remain undetected by conventional clinical risk stratification tools [14,16]. Resistance is driven by multiple aberrant signaling pathways (including BCR, PI3K/Akt, NF-κB, and JAK/STAT), along with clonal evolution and tumor microenvironmental influences—mechanisms that are not addressed within a molecularly agnostic therapeutic paradigm [17]. Multiple efforts to improve upon R-CHOP by means of treatment intensification or incorporation of targeted agents (R-DA-EPOCH, bortezomib, and lenalidomide) in large randomized trials have failed to demonstrate a meaningful survival benefit [16,18,19]. These findings underscore that universal treatment escalation, in the absence of molecular stratification, is insufficient to overcome resistance driven by tumor biological heterogeneity

A molecularly oriented strategy, grounded in tumor-specific molecular biomarkers, represents a promising and forward-looking solution for first-line therapy. This approach offers a means to overcome the key limitations of R-CHOP, as oncogenic signaling pathways vary substantially across genetic subtypes of DLBCL and directly govern tumor sensitivity to specific targeted therapies [7]. Incorporation of an additional therapeutic component guided by the tumor molecular profile allows for precise targeting of key resistance mechanisms, particularly in subgroups where standard R-CHOP demonstrates limited efficacy [20]. In this way, treatment transitions from a “one-size-fits-all” paradigm to a personalized strategy grounded in disease biology [21]. This rationale is supported by several international studies and clinical observations demonstrating successful application of such an approach [22,23,24].

Thus, investigating the effectiveness of differentiated therapy for newly diagnosed DLBCL is a timely and clinically relevant objective.

## 2. Materials and Methods

This study represents a continuation of a single-center, prospective, non-randomized interventional clinical trial aimed at personalizing first-line therapy for DLBCL based on mutational profiling.

Patients were included if they had superficially located, easily biopsy-accessible tumors (e.g., lymph node, gastric, or colonic biopsies) or sufficient archival formalin-fixed, paraffin-embedded (FFPE) tissue blocks for genotyping; if immediate life-saving treatment was not clinically indicated; and if they gave written informed consent to receive therapy per the adaptive protocol.

DLBCL diagnosis was confirmed by immunomorphological examination of tumor biopsy specimens in accordance with the criteria of the revised WHO classification of hematolymphoid neoplasms (2022) [3].

The clinical trial excluded patients with high-grade B-cell lymphoma, primary large B-cell lymphoma of immune-privileged sites (testis, central nervous system, and vitreoretinal areas), and primary mediastinal (thymic) large B-cell lymphoma.

To exclude high-grade B-cell lymphoma, fluorescence in situ hybridization (FISH) was performed to detect rearrangements of the *c-MYC*, *BCL2*, and *BCL6* genes.

DLBCL genotyping was performed according to the LymphGen classification.

Patients in the first cohort underwent Sanger targeted sequencing of a 19-gene panel: *MYD88*, *CD79B*, *TNFAIP3*, *EP300*, *PIM1*, *STAT6*, *NOTCH1*, *EZH2*, *CREBBP*, *TET2*, *NOTCH2*, *BTG1*, *CD70*, *TNFRSF14*, *DTX1*, *MPEG1*, *MTOR*, *TBL1XR1*, and *TP53*. The results convincingly demonstrated both the rationale and effectiveness of this diagnostic method for determining the genotype of DLBCL.

Since preliminary sequencing yielded encouraging results but revealed a high proportion of unclassified genotypes, we subsequently expanded the targeted lymphoid gene panel. Patients in the second cohort (“proof-of-concept”) underwent whole-exome sequencing of a 60-gene panel: *ARID1A*, *B2M*, *BTG1*, *BTG2*, *CCND3*, *CD70*, *CD79B*, *CIITA*, *CREBBP*, *DDX3X*, *DTX1*, *DUSP2*, *EP300*, *EZH2*, *FAS*, *GNA13*, *IRF4*, *IRF8*, *KMT2D*, *MPEG1*, *MYD88*, *NOTCH1*, *NOTCH2*, *PIM1*, *PRDM1*, *SGK1*, *SOCS1*, *STAT3*, *STAT6*, *TBL1XR1*, *TET2*, *TNFAIP3*, *TNFRSF14*, *TP53*, *ZFP36L1*, *MTOR*, *NFKBIA*, *ETV6*, *ACTG1*, *OSBPL10*, *C-MYC*, *BCL2*, *BCL6*, *FOXO1*, *ATM*, *CD79A*, *PIK3CD*, *PTEN*, *CD5*, *CD58*, *CDKN2A*, *CDKN2B*, *ASXL1*, *KRAS*, *EPHB1*, *BCL10*, *PRKCB*, *PLCG2*, *CARD11*, and *MEF2B.*

Initial staging was performed according to the Ann Arbor classification, modified by the Lugano 2014 criteria, including PET/CT and unilateral bone-marrow trephine biopsy [25]. If baseline PET/CT was not feasible due to disease severity, contrast-enhanced whole-body CT was performed at the onset.

Treatment response was assessed by PET/CT after cycles 2, 4, and 6 in accordance with the Lugano criteria. In cases with a Deauville score of 4–5 following cycle 4 or cycle 6, a repeat biopsy of the PET/CT-avid lesion was performed for histopathological (and molecular, when appropriate) confirmation of residual viable tumor. If the repeat biopsy after cycle 4 demonstrated no viable tumor, patients continued on the assigned protocol; if the repeat biopsy after cycle 6 demonstrated no viable tumor, patients completed protocol therapy. If viable tumor was confirmed at either time point, patients were transitioned to second-line therapy.

The treatment protocol included one induction cycle of ICT with R-CHOP as a bridge therapy pending receipt of molecular profiling results, followed by initiation of five induction cycles of R-CHOP-X (X—an additional antitumor agent). Depending on the identified genotype, patients received one of the agents designated as X: the first group received vorinostat, acalabrutinib, decitabine, or lenalidomide; the second group received acalabrutinib, decitabine, or lenalidomide (Table 1 and Table 2).

Treatment response was evaluated according to the International Lugano Criteria [26]. Adverse events were recorded by type and severity in accordance with the National Cancer Institute Common Terminology Criteria for Adverse Events (NCI CTCAE), version 5.0 [27].

Statistical analysis was performed using R software, version 4.2.2 (https://www.r-project.org; accessed on 12 September 2026). The analysis comprised descriptive and inferential components. Categorical variables are presented as percentages. Quantitative variables are presented as means ± standard deviations.

Kaplan–Meier methodology was used to generate PFS and OS curves. OS was calculated as the time from therapy initiation to death from any cause and was censored with the date of last available follow-up. PFS was calculated as the time from therapy initiation to disease progression, relapse, or death from any cause.

## 3. Results

### 3.1. Study Population and Disposition

Analysis of the study results was performed on 16 September 2025.

**Figure 1:**
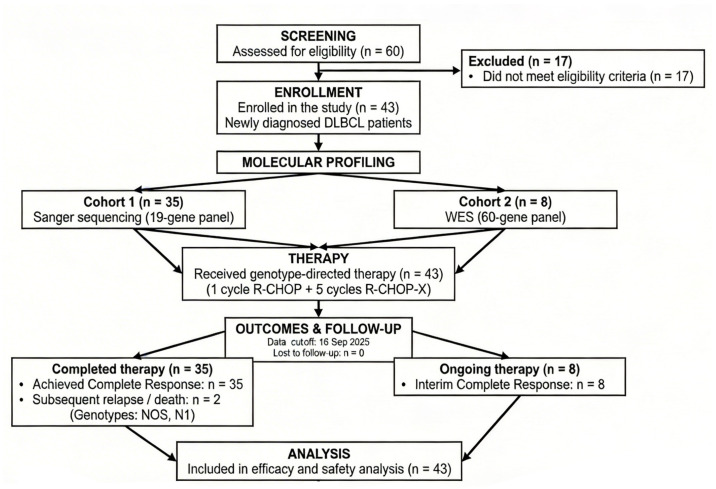
Study flow diagram detailing patient disposition, genotype-directed therapy, and clinical outcomes.

The detailed patient enrollment, LymphGen genotype-guided treatment assignment, and follow-up process are summarized in the study flow diagram in Figure 1.

### 3.2. Baseline Characteristics

The first and second cohorts included 35 and 8 patients, respectively, all with newly diagnosed DLBCL. The overall baseline characteristics of the patients are presented in Table 3 and Table 4.

Direct comparison of molecular profiling results obtained with two diagnostic panels underscores the clinical relevance of extended genomic testing. In the initial cohort analyzed with a narrow 19-gene panel using Sanger sequencing, a substantial proportion of patients (n = 12 (34%)) remained unclassifiable (NOS), reflecting the limited resolving power of the smaller panel. By contrast, in the “proof-of-concept” cohort assessed by whole-exome sequencing (WES) and analyzed using an expanded 60-gene panel, the proportion of NOS cases decreased markedly to only 12% (n = 1). This pronounced reduction supports the conclusion that a more comprehensive approach to mutational profiling substantially improves the accuracy and informativeness of the LymphGen diagnostic algorithm, thereby enabling genotype-directed targeted therapies for a larger number of patients.

### 3.3. Molecular Profiling and LymphGen Classification

To characterize the molecular landscape of the study population, we assessed pathogenic/likely pathogenic gene-level alterations together with FISH rearrangement status and assigned cases to LymphGen genetic subtypes. In Cohort 1 (n = 35), analyzed using the 19-gene Sanger sequencing panel, a heterogeneous distribution of genetic alterations was observed across cases, with corresponding classification into multiple LymphGen categories, including NOS, N1, EZB, MCD, BN2, and ST2. The detailed patient-level mutational profiles, FISH findings, and LymphGen assignments for Cohort 1 are presented in Table 5.

To further explore the utility of broader molecular testing, a proof-of-concept analysis was performed in Cohort 2 (n = 8) using the 60-gene WES panel. This cohort likewise showed molecular heterogeneity, with cases assigned to several LymphGen subtypes, including EZB, A53, ST2, N1, NOS, and MCD. The detailed patient-level mutational profiles, FISH findings, and LymphGen assignments for this cohort are summarized in Table 6.

### 3.4. Therapy Outcomes and Toxicity

The clinical outcomes of patients with newly diagnosed DLBCL are presented in Table 7.

All genetic subtypes initially attained a complete metabolic response (CMR). During the follow-up period, early disease relapse was observed in two patients harboring the NOS and N1 genetic subtypes. In both cases the relapse proved incurable and resulted in death due to progressive disease.

**Figure 2:**
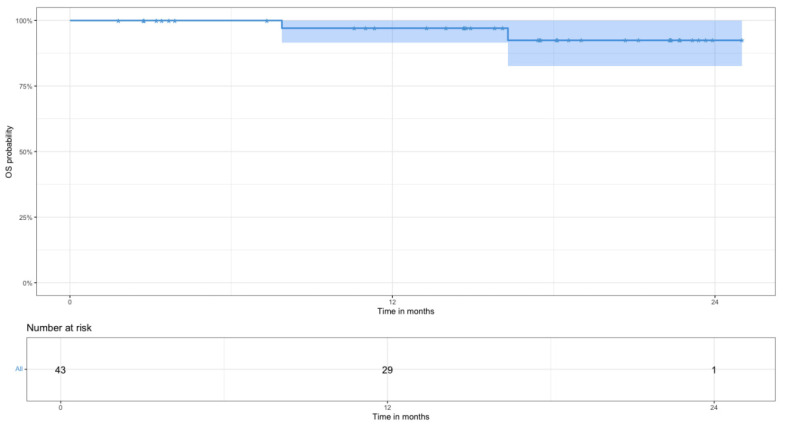
Two-year overall survival values with number at risk (n = 43).

**Figure 3:**
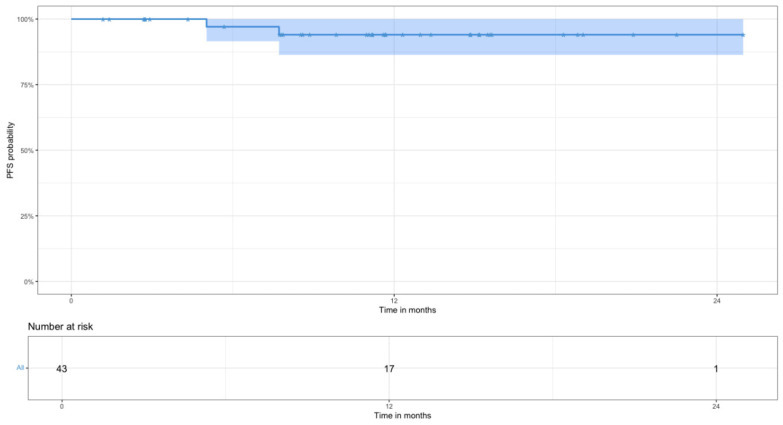
Two-year progression-free survival values with number at risk (n = 43).

Two-year OS and PFS for all patients with newly diagnosed DLBCL are shown in Figure 2 and Figure 3: 2-year OS was 92% (95% CI 83–100%) and PFS was 94% (95% CI 86–100%). 

The profile of hematologic toxicity is shown in Table 8. Non-hematologic adverse events were, in most cases, of mild to moderate severity (grade I–II).

## 4. Discussion

From a molecular biology perspective, DLBCL is an extremely heterogeneous disease. In recent years, the focus of clinical research has shifted toward personalized medicine, and DLBCL treatment is following this trend. Implementation of molecular diagnostics aimed at identifying biomarkers introduces a new stage in the management of newly diagnosed DLBCL.

The present clinical study is a unique project that, for the first time in the Russian Federation, implemented a personalized molecularly oriented approach to first-line therapy for newly diagnosed DLBCL. The analysis encompassed clinical and molecular–biological characteristics of the patients, direct efficacy, toxicity, and two-year PFS and OS.

The complete metabolic response in the analyzed cohort among those who completed therapy (n = 35) was 100%. Eight patients remained on treatment; the overall response rate is 100%. The two-year PFS and OS for the patients included in this work reached 94% and 92%, respectively, which is particularly important given the predominance of patients at high risk of early progression by IPI (3–5 points) in the studied cohort. According to data from large randomized trials, the 2-year PFS among patients in the high-risk IPI group (IPI score 3–5) treated with R-CHOP is approximately 65% [28,29].

The molecularly adapted treatment approach demonstrated a favorable safety and tolerability profile. At the analysis cutoff, no dose reductions, treatment delays, or early discontinuations had been recorded, permitting patients to complete the intended regimen on schedule or to continue therapy without protocol deviations.

As noted above, several fundamentally different strategies exist for treating newly diagnosed DLBCL.

The primary clinical tool used in routine practice to stratify patients by risk is the IPI. According to IPI, patients are divided into low-risk (0–2 points) and high-risk (3–5 points) groups. Considering results from studies of a risk-adapted approach, standard R-CHOP-21 is appropriate for patients at low risk of early progression, whereas Pola-R-CHP is recommended for high-risk DLBCL and, in some cases, planning for high-intensity induction therapy [30] may be considered.

Cellular therapies are actively entering frontline treatment for newly diagnosed DLBCL. Bispecific monoclonal antibodies directed against CD20xCD3 have demonstrated high efficacy in high-risk DLBCL as components of risk-adapted therapy. For example, epcoritamab combined with R-CHOP in a phase Ib/II study in patients with high-risk DLBCL according to IPI showed high efficacy: overall and complete metabolic response rates were 100% and 76%, respectively [31]. A randomized phase III trial is currently ongoing to evaluate the efficacy and safety of adding epcoritamab to R-CHOP in patients with high-risk DLBCL [32]. Similarly, the efficacy of another bispecific antibody, glofitamab, in combination with Pola-R-CHP is under investigation in the context of risk-adapted therapy [33].

Another approach is the response-adapted therapy strategy. Previous attempts to implement response-adapted strategies using intensified antitumor immunochemotherapy did not demonstrate a proven survival benefit. In the PETAL trial, patients after two cycles of R-CHOP with a PET/CT-positive interim response (ΔSUVmax < 66%) were randomized to two groups. The first one continued standard R-CHOP, while the second received a Burkitt-like intensified regimen. The results showed that therapy intensification did not improve survival but was associated with markedly increased toxicity [34]. Currently, the SAKK 38-19 study is evaluating whether adding acalabrutinib to R-CHOP after two cycles in patients with a PET/CT-positive interim scan and/or lack of a molecular response can improve clinical outcomes [35].

First-line CAR-T cell therapy has demonstrated high antitumor activity in patients with high-risk large B-cell lymphoma. The ZUMA-12 study design combined criteria of both a risk-adapted strategy (IPI ≥ 3) and response-adapted strategy (Deauville score 4–5 on interim PET/CT). The study enrolled 40 patients who received Axicabtagene Ciloleucel (axi-cel). Overall and complete response rates were 89% and 78%, respectively, while one-year PFS and OS were 74.6% and 90.6%. Median OS was 24.5 months [36]. A randomized phase III trial, ZUMA-23, is currently ongoing comparing axi-cel with standard therapy in high-risk patients (NCT05605899).

According to guideline recommendations, molecularly agnostic regimens, particularly R-CHOP, remain the standard of therapy for newly diagnosed DLBCL. However, the effectiveness of R-CHOP is markedly reduced in molecularly unfavorable DLBCL subtypes [10]. The totality of clinical and biological evidence supports a shift from molecularly agnostic to molecularly directed strategies based on the tumor’s genetic architecture.

In recent years, several pivotal clinical trials have evaluated the efficacy of molecularly targeted chemoimmunotherapy. In the randomized REMoDL-B trial published in 2019, adding bortezomib to standard R-CHOP did not confer a benefit in the studied cohort [16]. However, updated REMoDL-B results published in 2023 indicated that the bortezomib plus R-CHOP combination was more effective in ABC-DLBCL [37].

The randomized PHOENIX trial assessed the efficacy and safety of adding Ibrutinib, a Bruton tyrosine kinase inhibitor, to R-CHOP in patients with non-GCB DLBCL [38]. The trial did not meet its primary endpoint. A retrospective analysis that stratified patients by genetic subtype using the LymphGen algorithm, however, showed a survival benefit from Ibrutinib in younger patients with MCD and N1 genotypes of DLBCL (3-year event-free survival for those genotypes was 100%). Preclinical data had predicted an improved response in MCD but not in N1 [39].

In the randomized phase II GUIDANCE-01 trial, the R-CHOP-X approach—where the choice of an additional antitumor agent was dictated by genetic subtype according to LymphGen—produced statistically and clinically meaningful improvements in both short- and long-term outcomes versus R-CHOP [24]. The overall response rate was higher with R-CHOP-X compared with R-CHOP (92% vs. 73%, *p* = 0.005), and complete response rates were 88% versus 66% (*p* = 0.003). Two-year PFS was 88% in the R-CHOP-X group versus 63% in the R-CHOP (*p* < 0.001), and two-year OS was 94% versus 77% (*p* = 0.001).

The results of the present study are in close agreement with those of the GUIDANCE-01 protocol, thereby reinforcing the conceptual validity of the R-CHOP-X strategy as a biologically driven therapeutic paradigm. Importantly, however, our study extends this framework by incorporating a more granular approach to molecular stratification and a more mechanistically informed selection of targeted agents. In particular, application of the LymphGen algorithm enabled the identification of the ST2 subtype as a distinct biological entity, whereas ST2 was not reported as a separate molecular subtype in GUIDANCE-01. This distinction is not merely classificatory but has direct therapeutic implications, highlighting the importance of higher-resolution molecular profiling for precise patient allocation.

Notably, despite differences in diagnostic platforms, the therapeutic rationale demonstrated a high degree of convergence. Patients with ST2 and NOS subtypes in our cohort received lenalidomide, fully consistent with the treatment strategy applied to the NOS category in GUIDANCE-01, thereby providing indirect validation of the underlying biological assumptions. However, more substantive and conceptually important differences emerged in the management of the N1 subtype. While GUIDANCE-01 categorized this group as responsive to immunomodulatory therapy, our approach, informed by retrospective analyses of the PHOENIX study, reassigned N1—alongside MCD and BN2—to a BTK inhibitor-based strategy using acalabrutinib. This decision reflects a mechanistic reinterpretation of N1 biology, prioritizing BCR-dependent signaling and its interplay with NOTCH pathway alterations, and thus represents a more targeted attempt to overcome resistance in this subgroup.

In contrast, approaches to epigenetic modulation were fully concordant between the studies, further supporting the robustness of this therapeutic axis. The incorporation of vorinostat for the EZB subtype is consistent with the established class-specific activity of histone deacetylase inhibitors, as previously demonstrated for tucidinostat. Similarly, a clear therapeutic consensus was observed for *TP53*-mutated high-risk disease, where the use of decitabine in both studies underscores the central role of hypomethylating strategies in addressing primary resistance.

Collectively, these findings suggest that while the overarching R-CHOP-X paradigm is reproducible across studies, refinement of molecular classification and biologically informed therapeutic allocation may further enhance its clinical efficacy. This underscores the critical importance of integrating high-resolution genomic stratification into treatment decision-making as a prerequisite for fully realizing the potential of precision therapy in DLBCL.

Two ongoing multi-center, prospective, randomized trials are currently evaluating the efficacy and safety of personalized treatment approaches for newly diagnosed DLBCL: GUIDANCE-02 and GUIDANCE-05.

In GUIDANCE-02, all patients received a single induction cycle of standard R-CHOP after diagnosis, followed by tumor stratification according to molecular subtype using the LymphPlex classification and subsequent 1:1 randomization to complete the remaining five cycles either using R-CHOP-X or standard R-CHOP. Within the R-CHOP-X group, patients with MCD, BN2, or N1 subtypes received the BTK inhibitor Orelabrutinib in combination with R-CHOP; patients with EZB, ST2, or NOS subtypes received the immunomodulatory agent lenalidomide with R-CHOP; and patients harboring TP53 mutations received the hypomethylating agent decitabine combined with R-CHOP. The trial enrolled 1100 patients across 58 centers in China.

GUIDANCE-05 enrolled patients aged 18–75 years with newly diagnosed DLBCL and a IPI score of 2–5 who demonstrated a ≥3-log reduction in circulating tumor DNA (ctDNA) after one cycle of Pola-R-CHP. Following tumor stratification by genetic subtype using LymphPlex, patients will be randomized 1:1 to receive the subsequent five cycles using either Pola-R-CHP-X or standard Pola-R-CHP. Patients with MCD, BN2, or N1 subtypes will receive the BTK inhibitor Zanubrutinib in combination with Pola-R-CHP; those with EZB-MYC+/−, ST2, or NOS subtypes will receive lenalidomide with Pola-R-CHP; and patients with TP53 mutations will receive decitabine with Pola-R-CHP. The primary endpoint of this study is PFS.

Taken together, these trials test the hypothesis that combining distinct therapeutic modalities can improve outcomes in patients with newly diagnosed DLBCL. The most promising strategy appears to be an integrated approach that couples molecularly adapted therapy with response-adapted therapy based on dynamic ctDNA monitoring.

## 5. Limitations

This study has several limitations. First, it is a single-center, non-randomized study without a concurrent control group, which limits the ability to draw definitive causal conclusions. Second, the sample size is relatively small (n = 43), precluding robust subgroup analyses by individual LymphGen genotype. Third, two different sequencing approaches (19-gene Sanger panel and 60-gene WES panel) were used across cohorts, which may introduce classification heterogeneity. Fourth, the two-year follow-up, while encouraging, remains insufficient for definitive conclusions regarding long-term overall survival and late relapses. These findings warrant validation in larger, multi-center, randomized clinical trials.

## 6. Future Directions

Looking forward, the evolution of the precision medicine paradigm in DLBCL is expected to extend beyond static baseline molecular profiling toward the incorporation of dynamic monitoring tools, most notably serial assessment of circulating tumor DNA (ctDNA) and minimal residual disease (MRD). Early ctDNA clearance kinetics during first-line therapy have been shown to be a powerful predictor of clinical outcomes, enabling identification of patients at high risk of treatment failure well before the onset of clinical or radiographic progression. In this context, serial ctDNA analysis represents a highly promising biomarker platform for the development of response-adapted strategies, allowing for rational escalation or de-escalation of therapy.

Furthermore, in the setting of a rapidly expanding therapeutic landscape, integration of genotype-directed approaches with emerging cellular and immunotherapeutic modalities represents a particularly important area of investigation. The combined or sequential use of targeted agents with CAR-T cell therapies and bispecific monoclonal antibodies (CD20xCD3), such as epcoritamab and glofitamab, is of particular relevance for patients with biologically high-risk disease and an absence of early molecular response. Finally, despite encouraging results from pilot and single-center studies, the implementation of personalized, molecularly adapted strategies into routine clinical practice requires rigorous validation in large prospective, multi-center randomized trials.

## 7. Conclusions

Contemporary studies, including the present work, indicate that implementation of personalized therapy guided by the tumor mutational profile has the potential to substantially change the treatment paradigm for newly diagnosed DLBCL. This approach yields high antitumor efficacy and supports further optimization of therapy through precision-guided strategies.

## Figures and Tables

**Table 1:** R-CHOP-X treatment regimen in the first cohort.

Drug	Dosage and Route of Administration	Days of Administration	LymphGen Genotype
Rituximab	375 mg/m^2^, intravenously	0	-
Doxorubicin	50 mg/m^2^, intravenously	1	-
Vincristine	1.4 mg/m^2^, intravenously	1	-
Cyclophosphamide	750 mg/m^2^, intravenously	1	-
Prednisone	60 mg/m^2^, intravenously	1–5	-
Acalabrutinib	100 mg twice daily, orally	1–21	MCD, N1, BN2
Lenalidomide	25 mg once daily, orally	1–10	ST2, NOS
Vorinostat	400 mg once daily, orally	1–9	EZB
Decitabine	10 mg/m^2^, intravenously	1–5	A53

**Table 2:** R-CHOP-X treatment regimen in the second cohort.

Drug	Dosage and Route of Administration	Days of Administration	LymphGen Genotype
Rituximab	375 mg/m^2^, intravenously	0	-
Doxorubicin	50 mg/m^2^, intravenously	1	-
Vincristine	1.4 mg/m^2^, intravenously	1	-
Cyclophosphamide	750 mg/m^2^, intravenously	1	-
Prednisone	60 mg/m^2^, intravenously	1–5	-
Acalabrutinib	100 mg twice daily, orally	1–21	MCD, N1, BN2
Lenalidomide	25 mg once daily, orally	1–10	ST2, NOS
Decitabine	10 mg/m^2^, intravenously	1–5	A53, EZB

**Table 3:** Summary characteristics of patients (n = 35).

Parameters	Number of Patients, n (%)
Age (median), range (years)	63 (38–78)
ECOG performance status ≥ 2	24 (54)
Sex—male	15 (43)
IPI 3–5	28 (80)
Stage by Ann Arbor (Lugano modification, 2014)	
II	14 (40)
III–IV	21 (60)
Immunophenotype	
GCB	11 (32)
Non-GCB	24 (68)
Co-expression of c-MYC/BCL2	1 (3)
"Single-hit"	2 (7)
LymphGen:	
MCD	2 (6)
N1	11 (31)
BN2	2 (6)
EZB	5 (14)
A53	0 (0)
ST2	3 (9)
NOS	12 (34)

**Table 4:** Summary characteristics of patients (n = 8; “proof-of-concept”).

Parameters	Number of Patients, n (%)
Age (median), range (years)	68 (60–78)
ECOG performance status ≥ 2	1 (12)
Sex—male	4 (50)
IPI 3–5	3 (38)
Stage by Ann Arbor (Lugano modification, 2014)	
II	4 (50)
III–IV	4 (50)
Immunophenotype	
GCB	3 (38)
Non-GCB	5 (62)
Co-expression of c-MYC/BCL2	1 (12)
"Single-hit"	0
LymphGen:	
MCD	1 (12)
N1	1 (12)
BN2	0 (0)
EZB	2 (26)
A53	1 (12)
ST2	2 (26)
NOS	1 (12)

**Table 5:** Detailed mutational profiles and LymphGen genetic subtypes for patients in Cohort 1 (evaluated via the 19-gene Sanger sequencing panel, n = 35).

Patient	Detected Gene-Level Alterations (Pathogenic/Likely Pathogenic Variants)	FISH Rearrangements *(c-MYC, BCL2, BCL6*)	LymphGen Genotype
1	*PIM1*	None	NOS
2	*EP300, NOTCH2, TET2*	None	NOS
3	*EP300, NOTCH2*	None	NOS
4	*NOTCH2*	None	NOS
5	*EZH2, MYD88 p.L265P, NOTCH1, NOTCH2, TET2*	None	N1
6	*EP300, EZH2, STAT6, TNFRSF14*	*BCL2*	EZB
7	*EP300*	None	NOS
8	*CREBBP, EP300, MYD88 p.L265P, PIM1*	*BCL6*	MCD
9	*EP300*	*BCL6*	NOS
10	*NOTCH2*	None	NOS
11	*BTG1, NOTCH2, EP300, TBL1XR1*	*BCL6*	BN2
12	*CREBBP, DTX1, EP300, EZH2, MPEG1, NOTCH1, NOTCH2, PIM1, STAT6, TBL1XR1, TNFAIP3, TNFRSF14*	*BCL2*	EZB
13	None	None	NOS
14	*MYD88 p.L265P, PIM1, NOTCH2, MPEG1, MTOR*	None	MCD
15	*EP300, NOTCH1, NOTCH2, TBL1XR1, TET2*	None	N1
16	*EP300*	*C-MYC*	NOS
17	*BTG1*	*BCL6*	NOS
18	*EP300, NOTCH2, TBL1XR1*	*BCL2*	EZB
19	*NOTCH2*	None	NOS
20	*NOTCH2*	*C-MYC*	NOS
21	*EP300, NOTCH2, PIM1, SGK1, SOCS1*	None	ST2
22	*EP300, EZH2, STAT6, TET2, TNFRSF14*	None	EZB
23	*EZH2, NOTCH1, NOTCH2*	None	N1
24	*EP300, SGK1, SOCS1, TET2*	*BCL6*	ST2
25	*NOTCH1, NOTCH2, TNFRSF14*	None	N1
26	*CREBBP, STAT6, TET2*	*BCL2*	EZB
27	*TET2, TNFAIP3*	*BCL6*	BN2
28	*CD79b, CREBBP, NOTCH1, STAT6, TBL1XR1, TNFRSF14*	*BCL6*	N1
29	*CREBBP, NOTCH1, NOTCH2, TNFRSF14*	None	N1
30	*CD79b, NOTCH1, NOTCH2, MPEG1*	*BCL2*	N1
31	*CD79b, NOTCH1, NOTCH2, PIM1*	None	N1
32	*NOTCH1, DTX1, EZH2, TNFRSF14*	None	N1
33	*NOTCH1, TNFAIP3*	*BCL6*	N1
34	*CD79b, EP300, PIM1, NOTCH1, NOTCH2*	None	N1
35	*DUSP2, NOTCH2, TET2, TNFRSF14*	None	ST2

Note: Alterations are reported at the gene level (presence of pathogenic/likely pathogenic variants) according to the study sequencing workflow; variant allele frequency (VAF) was not available for systematic reporting. The canonical hotspot variant *MYD88 p.L265P* was specified when detected.

**Table 6:** Detailed mutational profiles and LymphGen genetic subtypes for patients in proof-of-concept Cohort 2 (evaluated via the 60-gene WES panel, n = 8).

Patient	Detected Gene-Level Alterations (Pathogenic/Likely Pathogenic Variants)	FISH Rearrangements (*c-MYC*, *BCL2*, *BCL6*)	LymphGen Genotype
1	*CREBBP, EP300, TNFRSF14*	None	EZB
2	*TP53, GNA13, TET2*	None	A53
3	*BTG2, DUSP2, SGK1, SOCS1*	None	ST2
4	*NOTCH1*	None	N1
5	*PIK3CD*	None	NOS
6	*MYD88 p.L265P, CD79b, KMT2D*	None	MCD
7	*TNFAIP3, CD79b, EP300, TNFRSF14*	None	EZB
8	*TET2, SGK1, PIK3CD*	None	ST2

Note: Alterations are reported at the gene level (presence of pathogenic/likely pathogenic variants) according to the study sequencing workflow; variant allele frequency (VAF) was not available for systematic reporting. The canonical hotspot variant *MYD88 p.L265P* was specified when detected.

**Table 7:** Outcomes of patients with newly diagnosed DLBCL, n (%).

Outcome	Completed Therapy (n = 35)	Ongoing Therapy (n = 8)
Overall response	35 (100)	8 (100)
Complete response	35 (100)	8 (100)

**Table 8:** The profile of hematologic toxicity, n (%).

Type of Toxicity	Number of Patients n = 43, (%)	Number of Cycles n = 242, (%)
Anemia grade 3–4	3 (7)	8 (3)
Thrombocytopenia grade 3–4	5 (12)	6 (2.5)
Neutropenia grade 3–4	12 (26)	14 (8)

## Data Availability

The data presented in this study are available on reasonable request from the corresponding author. The data are not publicly available due to ethical and privacy restrictions.

## Abbreviations

ABC	activated B-cell-like
BTK	Bruton tyrosine kinase
C-MYC	cellular myelocytomatosis oncogene
CT	computed tomography
DLBCL	diffuse large B-cell lymphoma
DUSP2	dual specificity phosphatase 2
FISH	fluorescence in situ hybridization
GCB	germinal center B-cell-like
NOS	not otherwise specified
OS	overall survival
PFS	progression-free survival
PET	positron emission tomography
WES	whole-exome sequencing
WHO	World Health Organization
